# A study on the role of measuring abdominal pressure in during weaning from mechanical ventilation

**DOI:** 10.1186/2197-425X-3-S1-A97

**Published:** 2015-10-01

**Authors:** MO Elghonemi

**Affiliations:** Cairo University, Critical Care, Cairo, Egypt

## Introduction

Increased Intra-abdominal pressure is common in critically ill patients [1] and it may adversely affect respiratory function. Mechanical ventilation (MV) is considered a predisposing factor for increased intra-abdominal pressure (IAP), especially when positive end-expiratory pressure (PEEP) is applied or in the presence of auto-PEEP. (2) However, it is not known if an increase in intra-abdominal pressure during weaning can be a marker for failure.

## Objectives

To assess the role of measuring intra-abdominal pressure in predicting success of spontaneous breathing trial, and whether intra-abdominal pressure can predicit risk of re-intubation in the first 24 hours after extubation.

## Methods

Study included 123 patients who were ventilated due to respiratory failure and were ready for weaning.To be enrolled in the study, patients had to have an improvement or resolution of the underlying cause of acute respiratory failure; adequate gas exchange as indicated by a PaO2 above 60 mm Hg while breathing with an Fi O2 of 0.40 or less with a positive end-expiratory pressure (PEEP) of 5 cm H2O or less; awake patients with a Glasgow Coma Scale score above 13; a temperature below 38° C; hemoglobin level above 10 g/dl; and no further need for vasoactive or sedative agents. In addition, the responsible physician had to agree that the patient was in stable condition and ready to be weaned from the ventilator. To measure IAP 50 ml of sterilized normal saline was inserted into the bladder using the Foley catheter and pressure was measured using the Krons technique. Baseline IAP was measured before SBT (IAP1)and was measured at 15 minutes interval for 60 minutes(IAP2-IAP3-IAP4-IAP5)Patients with BMI more than 30 ,abdominal pathology and baseline intrabdominal hypertension were excluded.

## Results

Out of123 patients,94 were successfully extubated after 60 minutes versus 29 who failed the SBT. Mean IAP at all time intervals was 7.4cmH20 in those who were successfully extubated versus 8.4 cmh20 in those who failed the SBT (p = 0.013). IAP was lower at all time intervals(IAP1,IAP2,IAP3,IAP4,IAP5) in patients who were extubated than those who failed (5.97,7.27,7.68,7.69,7.65 vs 6.79,11.28,10.92,15.33, 14.50) Out of the 94 patients who were successfully extubated, 31 patients were reintubated in the first 24 hours. The mean IAP in patients who were reintubated was 7.4 cmH20 versus 7.3 cmH20 in those who were not re-intubated.

**Figure 1:**
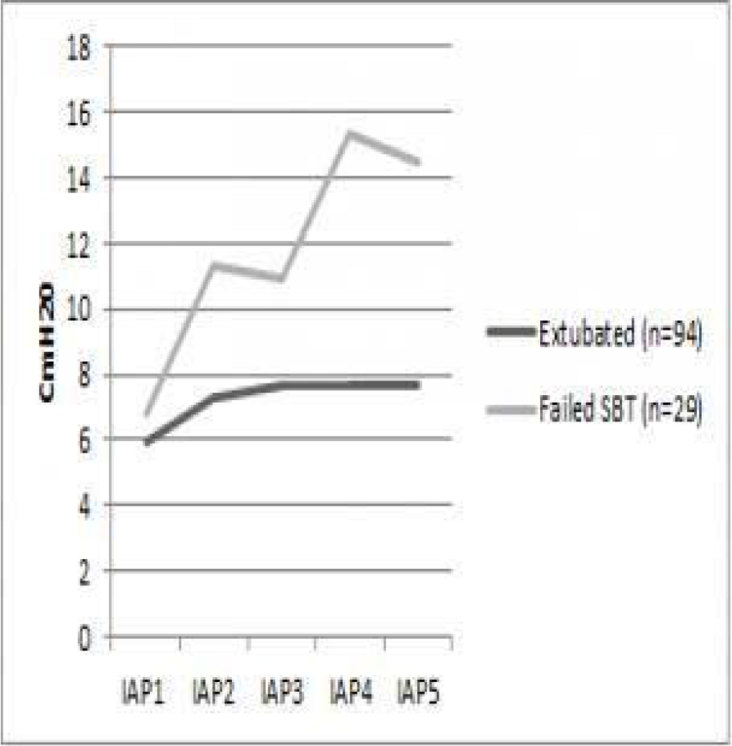
**IAP at different time intervals.**

**Table 1:** Mean IAP at Different Time Intervals.

	IAP1	IAP2	IAP3	IAP4	IAP5	
Extubated(n = 94)	5.97	7.27	7.68	7.69	7.65	
Failed SBT (n = 29)	6.79	11.28	10.92	15.33	14.55	

## Conclusions

Intrabdominal pressure is higher in patients who fail the Spontaneous Breathing trial druing weaning from Mechanical Ventilation and can be used to predicit failure. However, Intra-abdominal pressure was not significantly different in patients who were re-intubated in the first 24 hours after extubation.
